# Supplementary figures and images for: Aging boosts antiviral CD8+T cell memory through improved engagement of diversified recall response determinants
Source: PLoS Pathog. 2019 Nov 7;15(11):e1008144. doi: 10.1371/journal.ppat.1008144 (PMC6863560; doi:10.1371/journal.ppat.1008144)

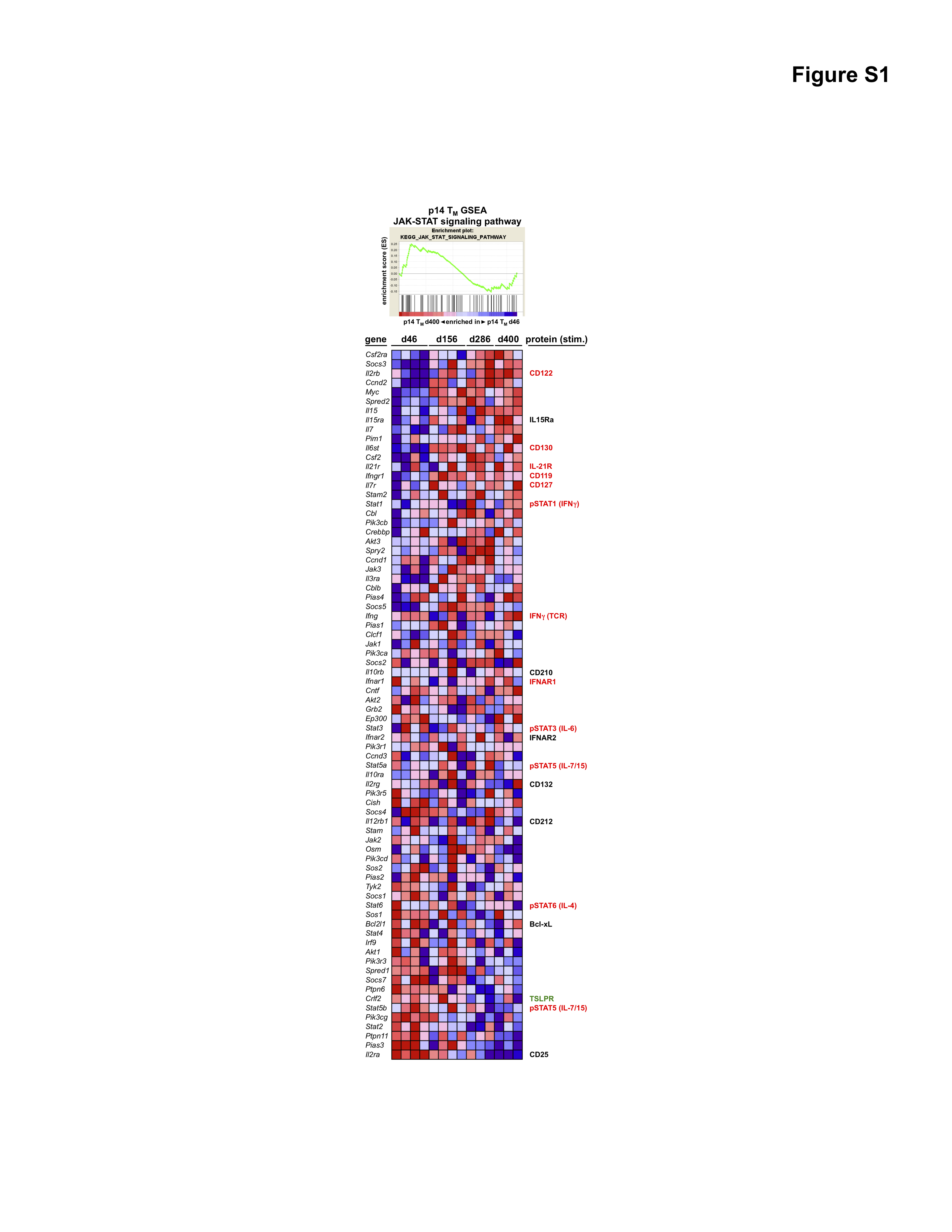

Supplement: S1 Fig — Time series GSEA were conducted with data sets obtained for aging p14 TM (d46, d156, d286 and d400) purified from LCMV-challenged p14 chimeras and processed directly ex vivo for microarray hybridization as detailed in refs.[9, 10]. Top: aged p14 TM are enriched for genes within the KEGG JAK-STAT signaling pathway module (normalized enrichment score: 1.05). Bottom: heat map displaying relative expression of individual genes by aging p14 TM (blue: low, red: high). The right hand column summarizes corresponding protein expression patterns conducted with aging DbNP396+ and/or DbGP33+ CD8+TM retrieved from spleen or blood of LCMV-immune B6 mice; colors identify significant expression changes accrued over time (red: upregulation; black: no change; green: downregulation); where indicated in parenthesis, CD8+TM were stimulated in vitro prior to analysis (IFNγ: 5h TCR stimulation with peptide; phosphorylated STAT proteins: 15min stimulation with indicated cytokines). The primary protein expression data in this summary are shown in Figs 2A/2B, 3A, S2 Fig and/or refs.[9, 10]. (TIF) [file ppat.1008144.s001.tif]

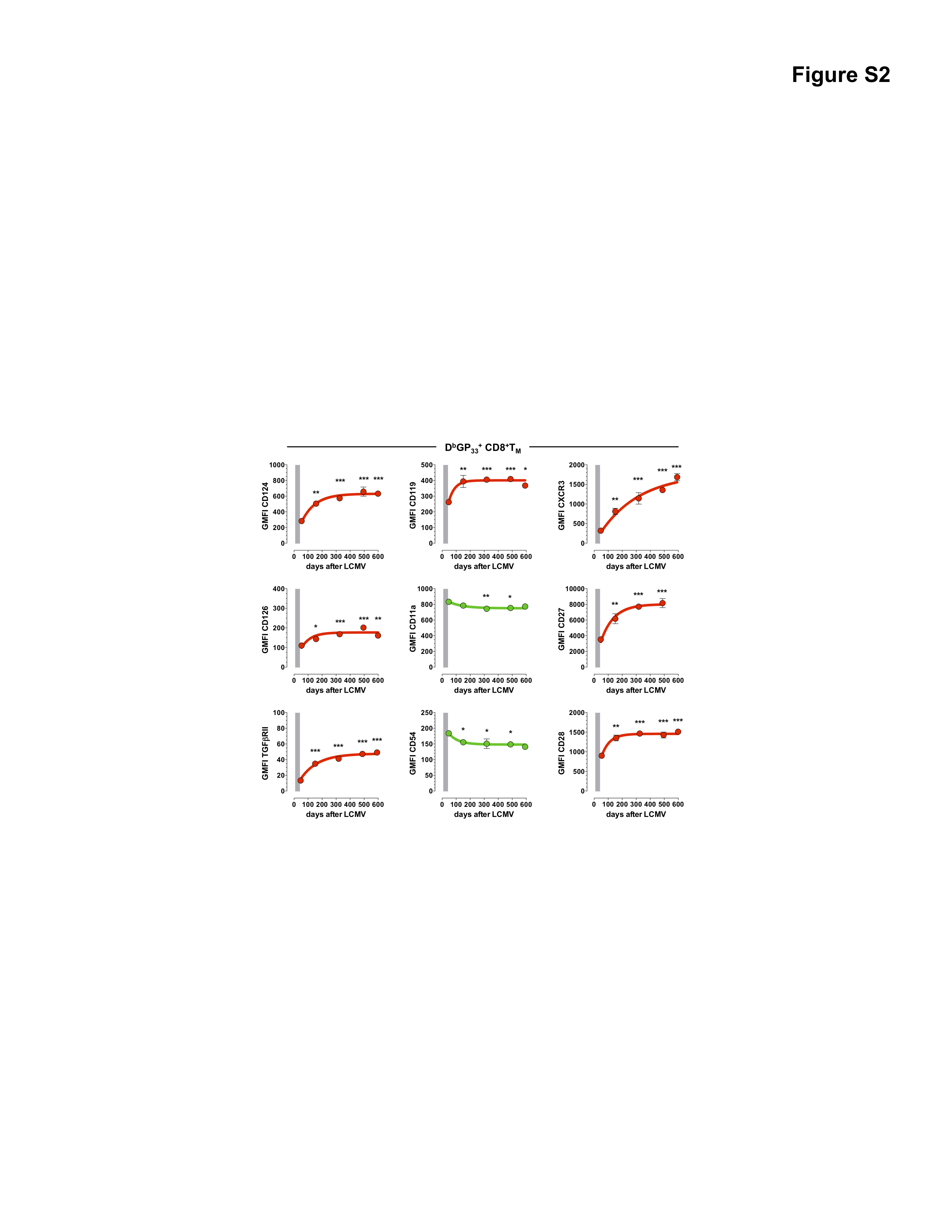

Supplement: S2 Fig — PBMC obtained from cohorts of aging LCMV-immune mice were contemporaneously stained to quantify expression levels of indicated receptors/ligands by DbGP33+ CD8+TM (GMFI: gometric mean of fluoresecence intensity; n≥3 mice per time point; statistical differences between young and older CD8+TM were calculated using one-way ANOVA with Dunnett’s multiple comparisons test). (TIF) [file ppat.1008144.s002.tif]

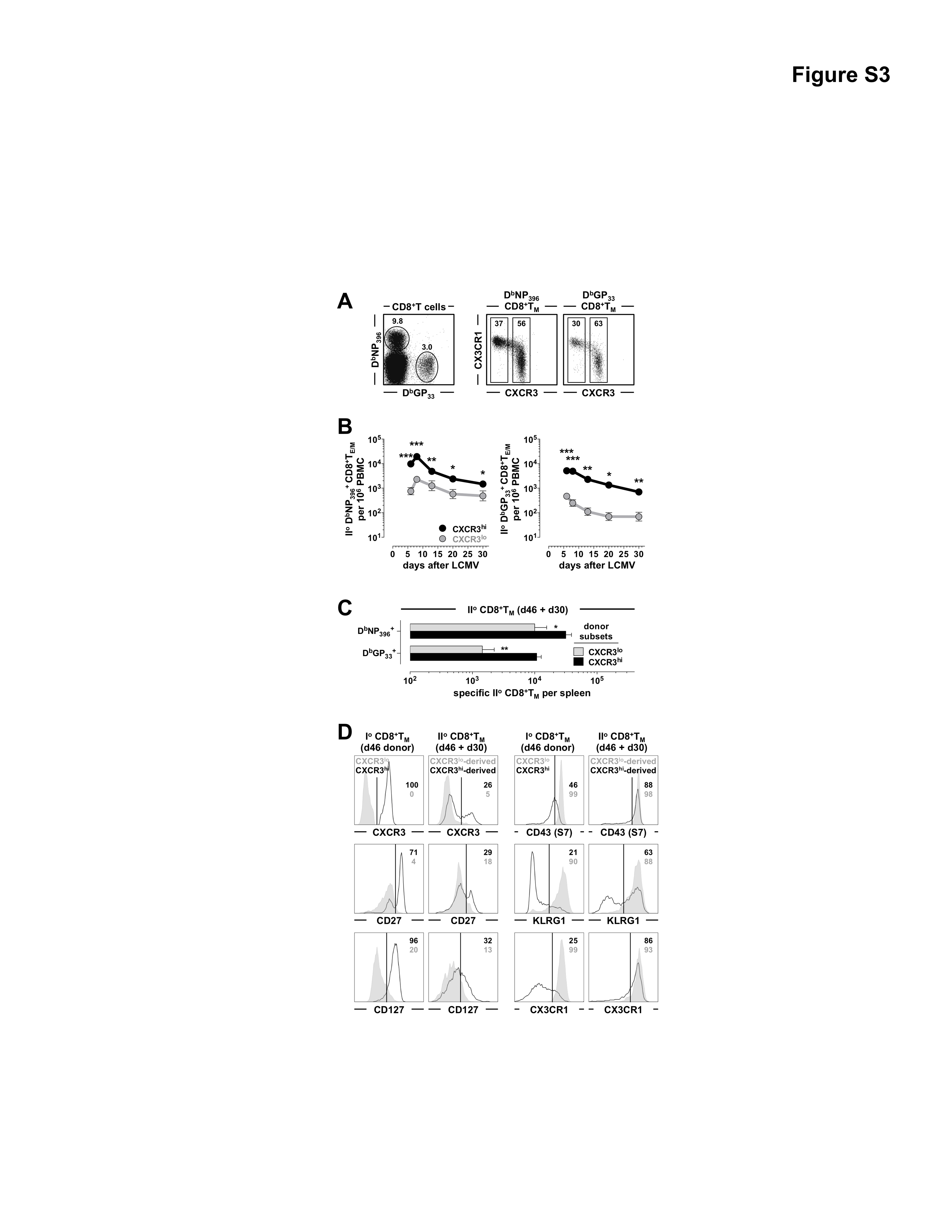

Supplement: S3 Fig — CXCR3hi and CXCR3lo CD8+T cell subsets were purified from young LCMV-immune B6.CD90.1 donors (d46) by combined magnetic bead and fluorescence activated cell sorting, and transferred i.v. into separate B6 recipients that were subsequently challenged with LCMV Arm (CXCR3hi transfers contained 5.0x10^3^ DbNP396+ and 1.2x10^3^ DbGP33+ CD8+TM per recipient, CXCR3lo transfers contained 5.0x10^3^ DbNP396+ and 1.35x10^3^ DbGP33+ CD8+TM per recipient). A., identification of DbNP396+ and DbGP33+ CD8+TM (left) and representative CXCR3 (and CX3CR1) expression pattern by DbNP396+ and DbGP33+ CD8+TM (right; the indicated regions demarcate CXCR3hi and CXCR3lo subsets corresponding to our sorting strategy). Values in dot plots are the percentage of cells within the indicated regions. B., kinetics of IIo DbNP396+ (left) and DbGP33+ (right) CD8+TE expansions as well as IIo CD8+TM development in peripheral blood (black: IIo CD8+TE/M derived from CXCR3hi donor CD8+TM, gray: IIo CD8+TE/M derived from CXCR3lo donor CD8+TM). C., specific IIo CD8+TM abundance in spleen (d30 after AT/RC); n = 5 mice per group and time point in panels B and C. D., histograms are gated on Io (donor, d46) or IIo (d46 + d30) DbNP396+ CD8+TM as indicated; the respective tracings correspond to the phenotypes of either CXCR3lo (gray filled) or CXCR3hi (black) Io donor CD8+TM subsets, or to those of IIo CD8+TM derived from CXCR3lo (gray) vs. CXCR3hi (black) Io CD8+TM (concatenated files of three mice/group; values featured in black or gray are the percentage of respective Io or IIo CD8+TM expressing high levels of indicated cell surface antigens). Note that CXCR3hi-derived IIo CD8+TM display a more mature phenotype as indicated by partial re-expression of high CXCR3, CD27 and CD127 levels as well as downregulation of CD43 (115kd glycoform), CX3CR1 and in particular KLRG1 (no differences were noted for CD62L and CD122 expression). (TIF) [file ppat.1008144.s003.tif]

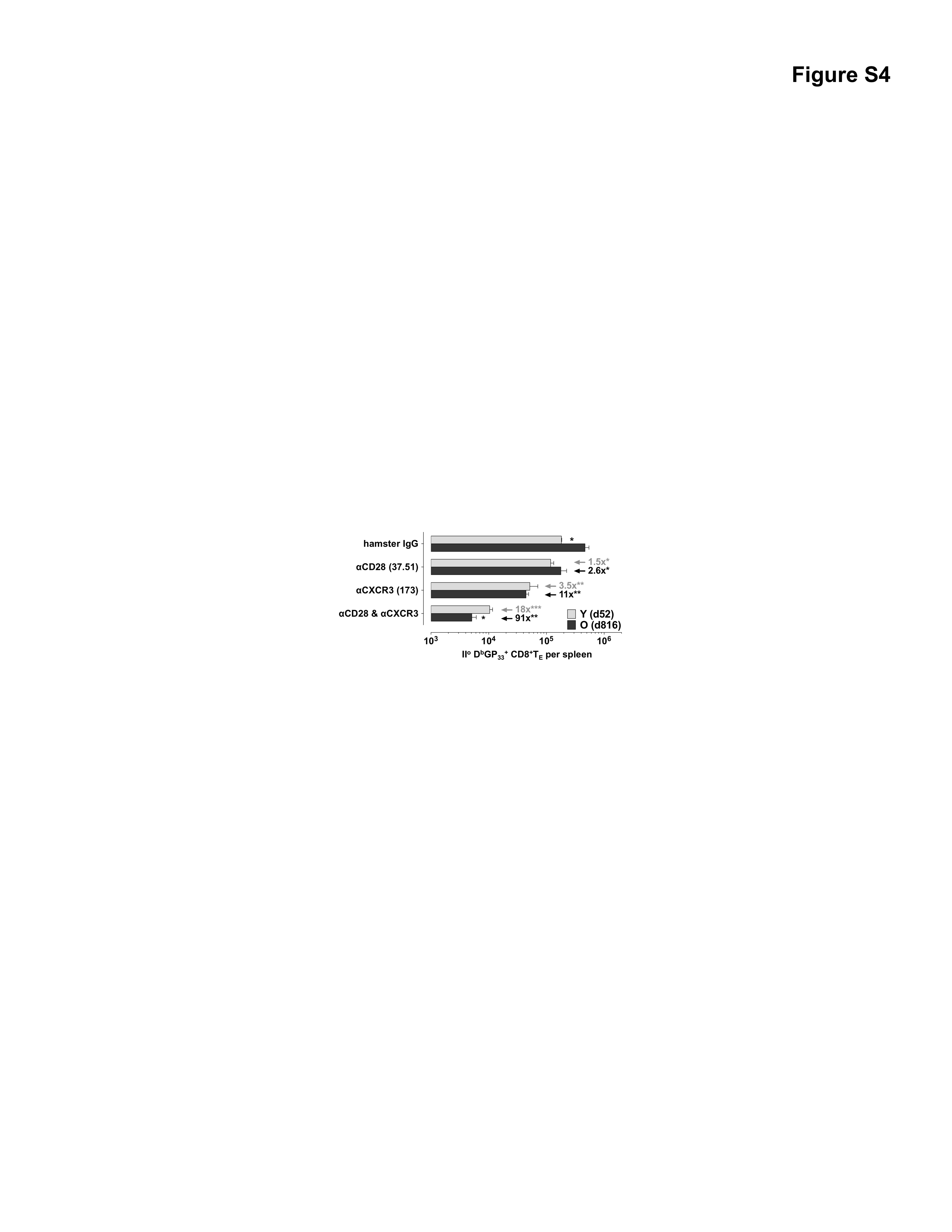

Supplement: S4 Fig — Mixed AT/RC Arm experiments were conducted under conditions of separate or combined CD28/CXCR3 blockade, and the bar diagram enumerates IIo DbGP33+ CD8+TE in the spleen with arrows, values and associated asterisks (significance) indicating the factors by which indicated treatment modalities reduced respective old and young recall responses in comparison to hamster IgG-treated control mice. While the overall reduction of IIo CD8+TE expansions after individual pathway blockade was somewhat less (αCD28 treatment) or more (αCXCR3 treatment) pronounced than in our other experiments (also evident at the level of impaired Io CD8+TE responses), combined CD28/CXCR3 blockade decreased old CD8+TM recall responses to a significantly greater extent than CD28 or CXCR3 blockade alone (p = 0.0204 and p = 0.0015, respectively); a similar synergism was also observed for the inhibition of young CD8+TM recall responses (αCD28/CXCR3 vs. αCD28 treatment p = 0.0022). These differences began to emerge in peripheral blood as early as d5 after AT/RC where only combination treatment resulted in a significant attenuation of aged (p<0.04) and to a lesser extent also young (p<0.04) IIo CD8+TE expansions (n = 3 mice/group and time point; AT of ~5x10^3^ young and old DbGP33+CD8+TM each). (TIF) [file ppat.1008144.s004.tif]
