# Supplementary material for: Aging boosts antiviral CD8+T cell memory through improved engagement of diversified recall response determinants
Source: PLoS Pathog. 2019 Nov 7;15(11):e1008144. doi: 10.1371/journal.ppat.1008144 (PMC6863560; doi:10.1371/journal.ppat.1008144)
Supplement: S1 Table — Details about all antibodies, staining dyes, magnetic beads, MHC-I monomers/tetramers, and recombinant cytokines used in the present study. (PDF) [file ppat.1008144.s005.pdf]

Table S1. Reagents & Materials

Name or antigen	Other name(s)	Antibody	Ab species/isotype	Format	Source
**Cell surface antibodies**					
CD3e		145-2C11	ahIgG1	FITC/PE/PerCP/eF450	BDBiosciences/ebioscience
CD4		RM4-5	rIgG2a	FITC/PE/PerCP/APC	BDBiosciences/ebioscience
CD8a		53-6.7	rIgG2a	FITC/PE/PerCP/APC/ BV605/BV786	BDBiosciences/ebioscience Biolegend
Integrin aL	CD11a	M17/4	rIgG2a	FITC/PE/APC	ebioscience
FcgR2	CD16/32	2.4G2	rIgG2b	purified ("Fc block")	BDBiosciences
Integrin b2	CD18	M18/2	rIgG2a	PE	ebioscience
CD25	IL-2Ra	3C7	rIgG2b	PE	BDBiosciences
TNFRSF7	CD27	LG.7F9	ahIgG	PE/PE-Cy7	ebioscience
CD28		37.51	shIgG	PE	ebioscience
CD43		S7	rIgG2a	FITC	BDBiosciences
CD44		IM7	rIgG2b	FITC/PE/APC	BDBiosciences/ebioscience
CD45.1		A20	mIgG2a	biotin/FITC/PE/APC	BDBiosciences
CD45.2		104	mIgG2a	FITC/PE/APC	ebioscience
ICAM1	CD54	YN1/1.7.4	rIgG2b	PE	ebioscience
CD62L		MEL-14	rIgG2a	APC-Cy7	ebioscience
CD90.1		OX-7	mIgG1	FITC/PerCP/BV650	BDBiosciences/Biolegend
		HIS51	mIgG2a	FITC/PE/APC	BDBiosciences/ebioscience
CD90.2		53-2.1	rIgG2a	FITC/PE/APC	BDBiosciences
ICAM2	CD102	3C4 (mIC2/4)	rIgG2a	FITC	Biolegend
CD119	IFNgRa/IFNgR1	2E2	ahIgG	purified/biotin/PE	Biolegend/ebioscience
		GR20	rIgG2a	biotin	BDBiosciences
CD122		5H4	rIgG2b	PE/BV650	Biolegend/BBDBiosciences
CD124	IL-4Ra	mIL4R-M1	rIgG2a	PE	BDBiosciences
CD126	IL-6Ra	D7715A7	rIgG2b	PE	Biolegend
CD127		A7R34	rIgG2a	BV711	Biolegend
CXCR3	CD183	49801	rIgG2a	PE	RnD Systems
		CXCR3-173	ahIgG	PE/APC/BV510	ebioscience/Biolegend
CX3CR1		SA011F11	mIgG2a	BV605	Biolegend
KLRG1		2F/KLRG1	shIgG	PE-Cy7/PerCP-Cy5.5	Biolegend
TGFbRII		polyclonal (FAB532P)	goat	PE	RnD Systems
**Intracellular antibodies**					
SAP (SLAM-associated protein)		12C4	rat	Alx647	A. Veillette
pSTAT1 (Y701)		4a	mIgG2a	Alx647	BDBiosciences
pSTAT3 (Y705)		4/P-STAT3	mIgG2a	Alx647	BDBiosciences
pSTAT6 (Y641)		J71-773.58.11	mIgG1	Alx647	BDBiosciences
**Isotpe controls**					
unknown		MOPC-21	mIgG1	FITC	BDBiosciences
Dansyl		27-35	mIgG2b	PE/APC	BDBiosciences
unkown		R35-95	rIgG2a	FITC/PE	BDBiosciences
unknown		A95-1	rIgG2b	FITC/PE	BDBiosciences
TNP		A19-3	ahIgG1	PE	BDBiosciences
unknown		G235-2356	ahIgG1	PE	BDBiosciences
***In vivo* treatment antibodies**					
CD4		GK1.5	rIgG2b	purified	R. Gill/BioXcell
CD11a	LFA-1	KBA	rIgG2a	purified	R. Gill
CD28		37.51	shIgG	purified	Biolegend/BioXcell
CD62L		MEL-14	rIgG2a	purified	Biolegend
TNFSF7	CD70	FR70	rIgG2b	purified	BioXcell
CD127	IL-7Ra	A7R34	rIgG2a	purified	P. Marrack/BioXcell
TNFSF5	CD154/CD40L	MR1	ahIgG	purified	R. Gill
CXCR3	CD183	CXCR3-173	ahIgG	purified	Biolegend
IFNγ		XMG1.2	rIgG1	purified	P. Marrack/R. Gill/BioXcell
IL-7		M25	mIgG2b	purified	P. Marrack/BioXcell
TGFβ1,2,3		1D11.16.8	mIgG1	purified	BioXcell
KLH		RTK2758	rIgG2a	purified	Biolegend
unknown		LTF-2	rIgG2b	purified	BioXcell
unknown		polyclonal (I4131)	rIgG	purified	Sigma
TNP/KLH		SHG-1	shIgG	purified	Biolegend
TNP/KLH		HTK888	ahIgG	purified	Biolegend
unknown		polyclonal (JOY000003)	hIgG	purified ("ChromPure")	Accurate Chemical
**Magnetic bead-conjugated antibodies**					
EasySep mouse CD8+ T cell enrichment kit (#19753)					StemCell Technologies
StemSep mouse CD8+ T cell enrichment kit (#13053)					StemCell Technologies

EasySep mouse PE positive selection kit (#18554)					StemCell Technologies
CD45R (B220) MicroBeads (#130-049-501)					Miltenyi Biotec
CD4 (L3T4) MicroBeads (#130-049-201)					Miltenyi Biotec
Anti-PE MicroBeads (#130-048-801)					Miltenyi Biotec
Dynabeads mouse pan B (B220) (#11441D)					Invitrogen/Dynal
**MHC-I monomers, SAv-fluorochromes, tetramers & viability dyes**					
DbNP396		n/a	n/a	biotin/APC	NIH Tetramer Core Facility
DbGP33		n/a	n/a	biotin	NIH Tetramer Core Facility
DbGP276		n/a	n/a	biotin/APC	NIH Tetramer Core Facility
Streptavidin	SAv	n/a	n/a	PE/APC<br>BV421	Invitrogen/Molecular Probes<br>Biolegend
Zombie Violet Fixable Viability Kit		n/a	n/a	n/a	Biolegend
**Dyes & probes**					
CFDA-SE		n/a	n/a		Invitrogen/Molecular Probes
**Recombinant cytokines**					
mIFNγ		n/a	n/a	recombinant, purified	Peprotech
mIL-4		n/a	n/a	recombinant, purified	Peprotech
mIL-6		n/a	n/a	recombinant, purified	Peprotech
mIL-10		n/a	n/a	recombinant, purified	Peprotech

n/a: not applicable; ah: Armenian hamster, m: mouse, r: rat, sh: Syrian hamster.
